# French Measles

**Published:** 1842-05

**Authors:** 


### FRENCH MEASLES.

We extract from the letter of A. B. Price, M. D., of Centreville, Ohio, dated April 25, 1842, the following notice:

"I have nothing of much interest to communicate, except that the Dengue (or as it is termed in common parlance, French Measles,) prevails to a considerable extent in this section of country. It is mostly a mild exanthematous disease. One case which occurred a few days ago, was attended with excruciating pain in the left foot and ankle, slight swelling, but no discoloration. The skin was so sensitive that the least touch of the part caused the patient to cry out. Before the eruption appeared, the pain extended to the knee and back; the pulse was not much affected, nor was there much febrile movement. The eruption comes out freely, about the third or fourth day; there is no affection of the throat, nor cough; eyes slightly inflamed; pulse but moderately accelerated; skin hot; head-ache; tongue only slightly furred; some thirst; restlessness, chilliness, and flying pains through the back and legs. Such are the symptoms of this singular epidemic; it attacks those who have had the measles, as well as those who have not. It has been almost confined to the young—appears to be contagious—treatment simple—unattended by danger."

